# Development of Tailored Composite Biopolymer Film Formulations Using Minimally Refined Chitosan from American Lobster (*Homarus americanus*) Shell Waste for Different Food Packaging Applications

**DOI:** 10.3390/polym17233132

**Published:** 2025-11-25

**Authors:** Abhinav Jain, Beth Mason, Marianne Su-Ling Brooks

**Affiliations:** 1 Department of Process Engineering and Applied Science, Dalhousie University, Halifax, NS B3H 4R2, Canada; abhinav.jain@dal.ca; 2 Verschuren Centre for Sustainability in Energy and the Environment, Sydney, NS B1M 1A2, Canada; bmason@verschurencentre.ca

**Keywords:** chitosan, gelatin, edible films, response surface methodology, active packaging, lobster shell waste, sustainable materials

## Abstract

The need for sustainable alternatives to petroleum-based plastic packaging has prompted interest in biodegradable biopolymer films. This study developed edible composite films using minimally refined chitosan from American lobster (*Homarus americanus*) shell waste combined with fish gelatin, glycerol, and sunflower oil. A Box–Behnken design within a response surface methodology (RSM) framework was used to investigate the effects of these formulation variables on ten key film properties, including mechanical strength, water sensitivity, barrier performance, and optical characteristics. High-quality empirical models (R^2^ ≥ 0.88) captured nonlinear, synergistic, and antagonistic interactions among the components, revealing trade-offs between competing attributes. Simultaneous multi-response optimization identified balanced formulations suited to various food packaging needs, including perishable, fresh, and dry products. Experimental validation of selected formulations confirmed model predictions within 5% error under laboratory conditions. Up to 68% of the inhibition activity against *Escherichia coli* was retained in a few composite formulations when compared with neat chitosan films, thus supporting their potential for active packaging. The key highlight of the present work is the use of crude chitosan derived from lobster shell waste, a low-cost, sustainable alternative to highly purified commercial sources, demonstrating the practical viability of marine byproduct valorization. Overall, this study advances the development of high-performance, application-specific biopolymer films and highlights RSM as an effective tool for optimizing multifunctional edible packaging materials. Future work should focus on enhancing antimicrobial functionality, evaluating real-world performance, and assessing consumer acceptance to support industrial adoption.

## 1. Introduction

The global plastic crisis has become an increasingly urgent environmental and public health concern. In 2023, global plastic production reached an estimated 414 million metric tonnes, marking a 3.3% rise from the previous year [1]. The packaging sector, being the primary driver of this output, accounts for approximately 40% of total plastic consumption and is the largest contributor to global plastic waste [2,3,4]. The continued and accelerating accumulation of this non-degradable waste in natural ecosystems has serious implications, including ecosystem degradation, microplastic bioaccumulation, and long-term human health risks, emphasizing the urgent need for sustainable alternatives to conventional petroleum-based plastics, particularly in food packaging where single-use products dominate [4,5].

Biopolymer-based biodegradable and edible films have gained attention as promising eco-friendly alternatives for reducing food packaging waste. Derived from natural macromolecules like polysaccharides and proteins, these materials offer a sustainable pathway to next-generation packaging solutions [6]. Among them, chitosan, a polycationic polysaccharide derived from chitin, stands out for its versatile functionality. Chitin is the second most abundant biopolymer after cellulose and is readily available in crustacean shell waste, making chitosan a bioresource with high accessibility [7]. Beyond being biodegradable and non-toxic, chitosan possesses excellent film-forming ability and broad-spectrum antimicrobial activity, making it highly attractive for food-contact applications [8].

In Atlantic Canada, the lobster (*Homarus americanus*) fishing and processing industry generates over 50,000 tonnes of chitin-rich shell waste annually. Currently, most of this biomass is underutilized or discarded into landfills and coastal waters due to a lack of valorization strategies [9]. Transforming this waste into high-value chitosan offers a significant economic opportunity for the coastal communities while also supporting a circular bioeconomy [10]. By leveraging this abundant marine byproduct, the development of chitosan-based films could address both regional waste management issues and the global demand for eco-friendly packaging alternatives.

Despite the desirable attributes associated with chitosan films, such as strong mechanical integrity and resistance to lipids and gases, films made solely from chitosan are often brittle, inflexible, and permeable to water vapor, limiting their independent use [11]. To overcome these constraints, researchers have explored various composite systems by combining chitosan with other compatible biopolymers [7,11]. In particular, proteins such as caseinate, whey, or gelatin have shown promise in effectively modulating critical film attributes like flexibility, thermal behaviour, and barrier properties [7,11,12,13].

Fish gelatin, a valorized protein derived from collagen-rich fish by-products, is a particularly promising partner for chitosan [14,15]. Films made from fish gelatin are naturally flexible and transparent, though prone to high water sensitivity and lack mechanical strength [14,16]. When combined with chitosan, the two polymers form polyelectrolyte complexes through electrostatic interactions between polycationic chitosan and the anionic groups in gelatin, yielding synergistic composite matrices [9]. For instance, the addition of chitosan to fish gelatin films has been shown to drastically reduce both water vapor permeability (WVP) and water solubility (WS) compared to gelatin films alone, creating a composite material with enhanced properties [14,16].

Additional tuning of film properties can be achieved by incorporating secondary additives. Glycerol is widely used as a plasticizer to improve film flexibility by increasing the mobility of polymer chains [17]. However, its hydrophilic nature may compromise vapour barrier properties [17,18]. To address the inherent water sensitivity of the chitosan matrix, a hydrophobic modifier can be introduced as well. While various readily available plant oils (e.g., olive or corn oil) can be effective in this context [19,20,21,22,23], sunflower oil was selected for the present study. Its choice was based on its wide commercial availability, low cost, and mild sensory profile, which aligns with the goal of using accessible, food-grade components. As a lipid, sunflower oil is intended to ‘boost water resistance’ and enhance film hydrophobicity. However, its incorporation presents a formulation challenge, as it can negatively affect mechanical strength or cause structural inconsistencies if not properly emulsified and dispersed [9,20]. This highlights the importance of careful formulation to achieve a balance between key film attributes.

The performance requirements for edible films are highly application specific. High-moisture foods like cheese require films with excellent water resistance and antimicrobial activity, while dry or instant foods may benefit from rapid water solubility and film disintegration [24,25]. A chitosan-gelatin film augmented with glycerol and oil presents a flexible platform to meet these diverse needs. However, the multifactorial nature of the system and complex interactions among ingredients necessitate a systematic optimization approach. Response Surface Methodology (RSM) is a robust statistical tool well-suited for this purpose. Unlike one-variable-at-a-time methods, RSM allows simultaneous optimization of multiple variables with fewer experiments, enabling the identification of nonlinear relationships, interaction effects, and formulation trade-offs [26].

The central hypothesis of the current work is that crude (unpurified) chitosan from lobster shells can be formulated into films with tunable functionality suitable for a range of food packaging applications. While several studies have explored chitosan–gelatin composite films, most rely on expensive, highly refined analytical-grade chitosan and lack comprehensive optimization of such multi-component formulations [27]. Building on earlier work from our laboratory demonstrating the compatibility and film-forming potential of minimally processed, crude lobster-shell chitosan combined with fish gelatin, glycerol, and locally sourced sunflower oil [9], the present study addresses these research gaps by employing a Box–Behnken RSM. This work systematically investigates the combined effects of chitosan–gelatin ratio, glycerol concentration, and sunflower oil content on key physicochemical, mechanical, optical, and barrier properties of the composite films. Predictive models were developed to identify optimal blends for targeted food packaging needs. Additionally, the antimicrobial activity of the optimized films was evaluated against *E. coli* to assess their potential for active packaging.

By integrating marine waste valorization with statistical modeling and functional performance evaluation, this study aims to demonstrate the potential and practical viability of using this composite system for developing sustainable packaging materials tailored for specific food applications.

## 2. Materials and Methods

### 2.1. Materials and Reagents

Crude chitosan was extracted from American lobster (*Homarus americanus*) shell waste supplied by Clearwater Seafoods Inc. (Bedford, NS, Canada), following the procedure described in our previous study [9]. Cold-water fish skin gelatin (Cat. no. G7041), glycerol, Tween 20, and glacial acetic acid were procured from Sigma-Aldrich (Oakville, ON, Canada). Commercial sunflower oil (Kernel brand) was purchased from a local superstore. Tryptic soy broth (TSB), Luria-Bertini (LB) broth, and agar were sourced from Fisher Scientific (London, ON, Canada). *Escherichia coli.* (ATCC 8739) was provided by the Verschuren Centre (Sydney, NS, Canada). All other chemicals and reagents were analytical grade.

### 2.2. Experimental Design

RSM with a three-factor, three-level Box–Behnken design (BBD) was used to study the combined effects of fish gelatin (Ge), sunflower oil (O), and glycerol (Gly) on critical performance variables of lobster-shell chitosan (LCh)-based composite films. The three independent variables and their levels were gelatin proportion (as % of total biopolymer mass, X_1_: 25, 50, 75% *w*/*w* polymer), sunflower oil concentration (X_2_: 0, 10, 20% *w*/*w* polymer), and glycerol concentration (X_3_: 0, 20, 40% *w*/*w* polymer). The total polymer content (LCh + Ge) was fixed at 2% *w*/*v* in all film-forming dispersions (FFD). These levels were set based on our preliminary findings in producing viable films.

A total of 17 experimental runs were conducted in a random sequence and included five centre point replicates. The coded BBD matrix and actual factor levels for each run are listed in Table 1. The data from each response variable (characterization parameters) were fitted to a second-order polynomial regression model (Equation (1)) using multiple linear regression and modeled as functions of the independent variables [28], and analysis of variance (ANOVA) was conducted. Contour and main effect plots were generated to visualize the influence of individual factors and their interactions on the responses.

(1) $$Y_{n}=\beta_{o}+\beta_{1}X_{1}+\beta_{2}X_{2}+\beta_{3}X_{3}+\beta_{12}X_{1}X_{2}+\beta_{13}X_{1}X_{3}+\beta_{23}X_{2}X_{3}+\beta_{11}X_{1}^{2}+\beta_{22}X_{2}^{2}+\beta_{33}X_{3}^{2}+\varepsilon$$

Here, Y_n_ is the predicted response; β_o_ is the intercept; β_1_ to β_33_ are regression coefficients; X_1_, X_2_, and X_3_ are the independent variables, and ɛ is the error term for the predicted response.

**Table 1:** Experimental design matrix for composite film formations.

Run	Coded Variables			Uncoded Variables		
	X_1_	X_2_	X_3_	Ge (X_1_) (% *w*/*w* Polymer)	O (X_2_) (% *w*/*w* Polymer)	Gly (X_3_) (% *w*/*w* Polymer)
1	−1	−1	0	25	0	20
2	+1	−1	0	75	0	20
3	−1	+1	0	25	20	20
4	+1	+1	0	75	20	20
5	−1	0	−1	25	10	0
6	+1	0	−1	75	10	0
7	−1	0	+1	25	10	40
8	+1	0	+1	75	10	40
9	0	−1	−1	50	0	0
10	0	+1	−1	50	20	0
11	0	−1	+1	50	0	40
12	0	+1	+1	50	20	40
13	0	0	0	50	10	20
14	0	0	0	50	10	20
15	0	0	0	50	10	20
16	0	0	0	50	10	20
17	0	0	0	50	10	20

Ge: fish gelatin proportion; O: sunflower oil content; Gly: glycerol content.

### 2.3. Preparation of the Films

Films were prepared via a casting method adapted from previous studies, with slight modifications [9,26]. A 2% (*w*/*v*) LCh solution was prepared by dissolving LCh powder in 1% (*v*/*v*) aqueous acetic acid under continuous stirring at 60 °C for three hours. Fish gelatin (Ge) solution at 2% (*w*/*v*) was prepared by hydrating gelatin powder in distilled water at 4 °C for 30 min, followed by dissolution at 60 °C for one hour. LCh–Ge blends were prepared by mixing appropriate volumes of the LCh and Ge solutions to achieve the targeted polymer ratios defined by the BBD matrix, maintaining a total polymer concentration of 2% (*w*/*v*). For formulations containing sunflower oil and glycerol, their specified amounts were added to the polymer blends. Tween 20 (30% *w*/*w* of oil) was used as an emulsifier in oil-containing formulations. Each film-forming dispersion (FFD) underwent homogenization and degassing with a probe sonicator (VCX 750, Vibra-Cell™, Sonics and Materials, Newtown, CT, USA) at 40% amplitude with 15-s pulses for 10 min. Approximately 0.25 g/cm^2^ of each FFD was then cast into Petri dishes (100 × 15 mm) and placed in a water-jacketed incubator (Forma Scientific 3250, Fisher Scientific, Champaign, IL, USA) for drying at 37 °C for three days. The resultant films were removed from the dishes and conditioned by placing them in a desiccator containing saturated magnesium nitrate solution (52–54% relative humidity) at 21 ± 2 °C for a minimum of three days before characterization.

### 2.4. Characterization of Prepared Films

#### 2.4.1. Film Thickness (FT)

The thickness of each film was measured using a digital micrometer (Neoteck^®^, New York, NY, USA) with a precision of 0.001 mm. To account for spatial variation, ten random points were measured across the film surface, and the mean value was recorded and used for subsequent calculations of other film properties.

#### 2.4.2. Equilibrated Moisture Content (EMC)

Circular samples (20 mm diameter) from conditioned films were punched using a precision punch tool (Boehm^®^, Hoffmann Group, Knoxville, TN, USA). The initial weights were recorded, followed by drying at 103 °C in a hot-air convection oven for 24 h, or until constant weight was reached. The EMC was then calculated as the percentage difference between the initial and final weights.

#### 2.4.3. Degree of Swelling (DS) and Water Solubility (WS)

The degree of swelling (DS) and water solubility (WS) for the films were determined based on the existing methods [29], with minor modifications. Pre-weighed dry film discs (20 mm diameter) were immersed in 15 mL of distilled water and agitated at room temperature for 24 h. After immersion, excess surface water was removed from the swollen samples by blotting, and the samples were immediately weighed. DS was calculated from the percentage increase in mass of the undissolved portion. The blotted samples were then re-dried at 103 °C to constant weight, and WS was computed as the percentage mass loss relative to the initial dry weight.

#### 2.4.4. Water Vapor Permeability (WVP)

Water vapor permeability (WVP) was measured gravimetrically based on ASTM E96/E96M-16 [30], with slight adjustments. Film samples were sealed over circular openings (2.8 cm^2^) of glass vials containing 10 mL of distilled water to maintain 100% relative humidity (RH) inside. The vials were placed in desiccators containing silica gel to maintain 0% RH at 22 ± 2 °C. The mass loss from each vial was recorded at regular intervals using a balance with 0.1 mg precision over seven days. WVP was calculated by first determining the water vapor transmission rate and then adjusting for film thickness and vapor pressure gradient, including correction for stagnant air resistance [31].

#### 2.4.5. Surface Hydrophobicity

The surface hydrophobicity of the films was characterized by contact angle (CA) measurements using ethylene glycol as the probing liquid [22]. A 20 µL drop was deposited on the film surface, and CA was recorded within 20 s using a drop shape analyzer (DSA25B, Krüss GmbH, Hamburg, Germany). Each film was measured at three different locations to obtain a reliable average.

#### 2.4.6. Film Opacity (OP)

Film opacity in the ultraviolet and visible light regions was evaluated using a UV-Vis microplate reader (Infinite M1000 Pro, Tecan, Männedorf, Switzerland) [9,32]. Circular film samples (5 mm diameter) were placed in a 96-well transparent microplate, and absorbance spectra were recorded between 230 and 800 nm. Opacity indices in the UV (OP_UV_: 230–400 nm) and visible (OP_VIS_: 400–800 nm) ranges were calculated by integrating the area under the absorbance curve and normalizing by the average film thickness [32]. The results were reported in units of A·nm/mm.

#### 2.4.7. Mechanical Properties

Tensile strength (TS), elongation at break (%EAB), and elastic modulus (EM) were assessed using a universal testing machine (EZ Test EZ-LX HS, Shimadzu, Kyoto, Japan) with a 1 kN load cell, following ASTM D882-18 [33]. Film strips (70 × 6 mm) were cut and conditioned prior to testing. Six replicates for each formulation were mounted with an initial grip separation of 30 mm, and tensile testing was done at a crosshead speed of 12.5 mm/min. The Trapezium-X software Version 1.5.3 (Shimadzu, Japan) was used to analyze the stress–strain data and calculate the mechanical parameters.

### 2.5. Simultaneous Optimization and Validation

To achieve edible films with an optimal balance of physical, mechanical, barrier, and optical properties, a simultaneous multi-response optimization was conducted using the desirability function approach within the RSM framework in Minitab Statistical Software Version 19. Each target response (e.g., TS, %EAB, WVP, WS, CA) was transformed into a unitless desirability value (*d_i_*), ranging from 0 to 1. These individual desirability values were then geometrically aggregated to yield an overall desirability score (*D*). Equal weights were assigned to each response to ensure a balanced optimization, with response targets tailored to the functional requirements of specific food packaging applications.

The optimal formulation was selected based on the highest overall desirability index (*D*). To validate the RSM outputs, three films with predicted optimal compositions were then prepared following the same procedures described in Section 2.3. These validation films were characterized for all relevant properties and compared with the model predictions. Predicted values were considered valid if the experimental results fell within the 95% confidence interval.

### 2.6. Antimicrobial Testing

The antimicrobial efficacies of three selected films were evaluated against *Escherichia coli* ATCC 8739 using a modified broth culture method adapted from existing methods [34,35]. Approximately 50 mg samples of the UV-sterilized films were introduced into 25 cm^2^ T-flasks containing 10 mL of tryptic soy broth (TSB). Sterile low-density polyethylene (LDPE) films served as negative controls, while commercial ampicillin discs (10 µg, BD BBL™ Sensi-Disc™) served as positive controls.

Each flask was inoculated with mid-log phase *E. coli* at ~10^6^ CFU/mL and kept at 37 °C with constant shaking for 12 h. Bacterial growth was monitored at 6, 12 and 24 h by measuring optical density at 600 nm (OD_600_). In addition, 20 µL of these TSB suspensions at 24 h were sub-cultivated on LB agar plates. The percentage inhibition of bacterial growth was calculated relative to the LDPE control. All experiments were performed in duplicate.

### 2.7. Statistical Analysis

Minitab Statistical Software (Version 19, Minitab Inc., State College, PA, USA) was used for experimental design, statistical analyses and optimization. Evaluation of the statistical significance of models and individual terms was done at a significance level of 1% or 5%. Physicochemical and antimicrobial data were analyzed using one-way ANOVA and Tukey’s HSD post hoc test, with *p* < 0.05 considered statistically significant. Data are reported as mean ± standard deviation (SD).

## 3. Results and Discussion

### 3.1. RSM Model Fitting

The mean values of each response variable for all tested film formulations are summarized in Supplementary Table S1. All eleven response variables evaluated, i.e., FT, EMC, DS, WS, WVP, CA, OP_UV_, OP_VIS_, TS, %EAB, and EM, were successfully fitted to full second-order polynomial models as a function of the formulation variables (Ge, O, and Gly content). Analysis of variance (ANOVA) was performed to evaluate statistical significance, and the F-values associated with each model and its terms are shown in Table 2. All derived second-order models were found to be statistically significant (*p* < 0.05), with many demonstrating high significance at the 99% confidence level (*p* < 0.001), indicating that the chosen variables had a substantial impact on the film properties. The results revealed that most responses were influenced by both linear and quadratic terms of the independent variables, and select responses also showed significant interaction effects between variables, underscoring the complex nature of component interplay. Importantly, for all models, the lack-of-fit was non-significant (*p* > 0.05), confirming that the selected polynomial models adequately described the observed variation in the data.

To eliminate overfitting, non-significant terms (*p* > 0.05) were eliminated from the full models to yield reduced polynomial models for each response (Equations (2)–(12)) [26]. The coefficient of determination (R^2^), adjusted R^2^ (adj-R^2^), and predicted R^2^ (pred-R^2^) for these reduced models are summarized in Supplementary Table S2. All reduced models demonstrated strong statistical performance, with high R^2^ values ranging from 0.88 to 0.97, indicating that at least 88% of the variability in the response data was captured by all the models. Adj-R^2^ values ranged from 0.83 to 0.96, and pred-R^2^ ranged from 0.70 to 0.95, further confirming the models were both statistically sound and had strong predictive power [36].

(2) $$Y_{FT}=58.37-0.153X_{1}+0.763X_{2}+0.627X_{3}+0.044X_{2}^{2}-0.006X_{1}X_{3}-0.015X_{2}X_{3}$$

(3) $$Y_{EMC}=21.52-0.452X_{1}-0.209X_{2}+0.159X_{3}+0.004X_{1}^{2}+0.008X_{3}^{2}$$

(4) $$Y_{DS}=187.3+0.593X_{1}-5.090X_{2}-8.729X_{3}+0.122X_{3}^{2}+0.146X_{2}X_{3}$$

(5) $$Y_{WS}=0.20+1.0551X_{1}-0.065X_{2}-0.236X_{3}-0.008X_{1}^{2}+0.003X_{3}^{2}-0.007X_{1}X_{2}-0.003X_{1}X_{3}+0.010X_{2}X_{3}$$

(6) $$Y_{WVP}\times 10^{-3}=944-5.0X_{1}+38.3X_{2}+10.79X_{3}-2.529X_{2}^{2}+0.643X_{3}^{2}$$

(7) $$Y_{CA}=45.4+0.682X_{1}+2.560X_{2}-0.293X_{3}-0.010X_{1}^{2}-0.116X_{2}^{2}$$

(8) $$Y_{OPUV}=1350-11.23X_{1}+203.0X_{2}-5.604X_{2}^{2}$$

(9) $$Y_{OPVIS}=603-11.44X_{1}-9.02X_{2}+0.116X_{1}^{2}+0.372X_{1}X_{2}$$

(10) $$Y_{TS}=86.2+0.305X_{1}-0.518X_{2}-2.929X_{3}-0.012X_{1}^{2}+0.029X_{3}^{2}+0.017X_{1}X_{3}$$

(11) $$Y_{EAB}=11.77+1.953X_{3}$$

(12) $$Y_{EM}=2292-6.97X_{1}-12.01X_{2}-66.08X_{3}+0.559X_{3}^{2}$$

Both the linear and quadratic terms for all three independent formulation variables were statistically significant (*p* < 0.05) for most responses. Gelatin proportion (X_1_) had the most pronounced effect across responses, significantly influencing mechanical properties (TS and EM), physical properties (FT, EMC, DS, WS) and surface hydrophobicity (CA). Sunflower oil concentration (X_2_) significantly contributed to changes in optical, hydrophobic and barrier-related properties (OP_VIS_, CA, WS, and WVP). Glycerol concentration (X_3_) primarily affected moisture-related responses and flexibility (%EAB). Several interaction terms, particularly X_1_·X_2_ (Ge–O) and X_1_·X_3_ (Ge–Gy), also showed significant effects on TS, WS, and barrier properties, suggesting synergistic or antagonistic interactions between components within the film matrix. These findings align with previous RSM optimization studies on chitosan-based composite films, which also reported strong predictive capabilities for second-order polynomial models when evaluating multicomponent edible films [37,38]. The significant model fits in the present study are consistent with the literature and validate the suitability of RSM for complex multicomponent biopolymer systems.

### 3.2. Influence of Formulation Variables on Physical Properties

**Figure 1:**
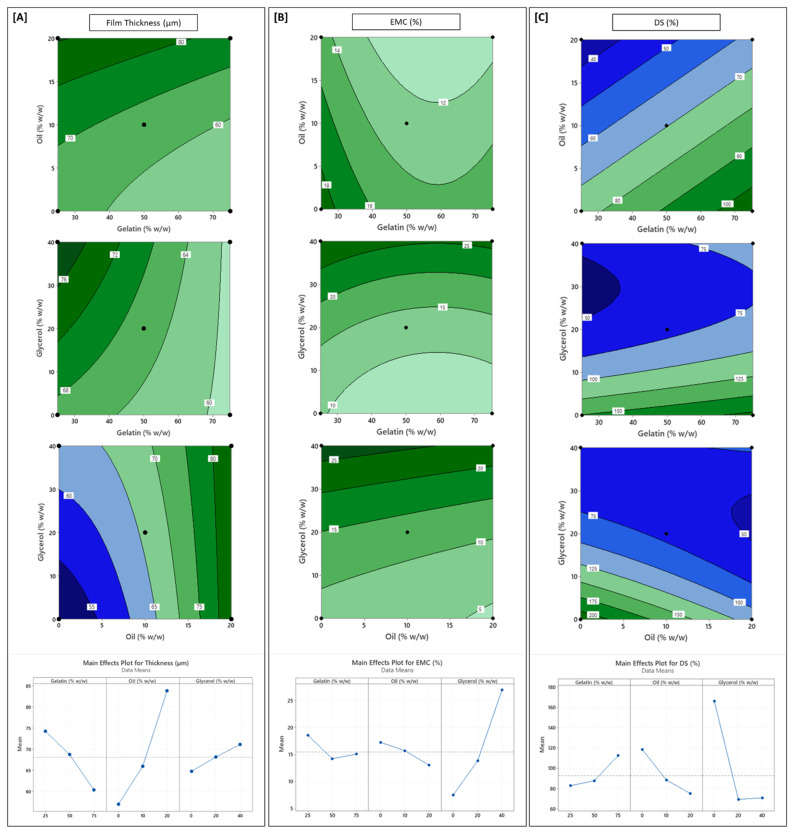
Contour and main effect plots for (**A**) film thickness (FT); (**B**) equilibrated moisture content (EMC); (**C**) degree of swelling (DS). In each case, the third variable was fixed at its midpoint value to generate the contour plots.

**Figure 2:**
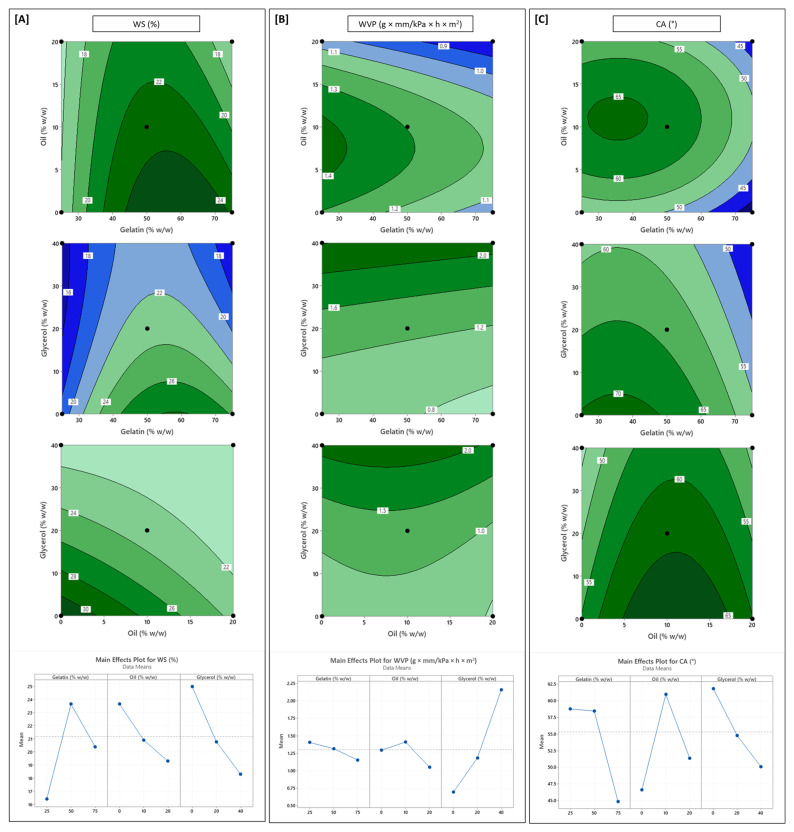
Contour and main effect plots for (**A**) water solubility (WS); (**B**) water vapor permeability (WVP); (**C**) surface contact angle (CA). In each case, the third variable was fixed at its midpoint value to generate the contour plots.

The physical properties of chitosan-based films, i.e., FT, EMC, DS, and WS, are crucial indicators of their functionality in food packaging and edible applications. These properties not only influence film integrity and handling but also govern moisture barrier performance, responsiveness to aqueous environments, and structural stability under varying environmental conditions. The derived predictive models for all four physical responses showed high statistical reliability (R^2^ > 0.94; adj-R^2^ > 0.91; pred-R^2^ > 0.78). Figure 1 and Figure 2 present the response surface contour plots and the main effects plots for each physical response investigated.

Film thickness (FT), which ranged from approximately 45 µm to 90 µm, was significantly influenced by all three independent variables (Equation (2)). Sunflower oil concentration (X_2_) was the most dominant factor, exhibiting a strong positive non-linear relationship with FT. Glycerol (X_3_) also demonstrated a significant positive linear effect, while gelatin proportion (X_1_) had a negative linear influence (Figure 1A). The increase in FT with rising oil and glycerol levels can be primarily attributed to the contribution of these components to the overall dry matter and higher solids content per unit area in the FFDs. Additionally, glycerol and oil can disrupt the dense packing of chitosan and gelatin chains by increasing intermolecular spacing and promoting matrix relaxation during drying [18,20]. These effects are consistent with previous findings in plasticized or lipid-modified chitosan films [18,21,39]. In contrast, increasing the gelatin proportion resulted in thinner films. Gelatin, due to its conformational flexibility and reduced steric hindrance, can facilitate denser packing of the chitosan-gelatin matrix [32,40], resulting in more compact structures.

Significant interaction effects were also observed for FT. For example, the gelatin–glycerol (X_1_·X_3_) interaction (*p* < 0.05) suggests that the thickening effect of glycerol diminished in gelatin-rich films, potentially due to restricted migration of glycerol into tightly packed matrices. Similarly, the oil–glycerol (X_2_·X_3_) interaction (*p* < 0.05) implies a moderate thickening effect of glycerol when the bulkier oil molecules were present in the matrix at higher concentrations. This could be due to molecular competition in disrupting polymer chain interactions, or limitations in the degree of expansion possible in the film once already bulked by oil.

Equilibrated moisture content (EMC), which ranged from 4.0% to 31.4%, was strongly governed by the hydrophilic–hydrophobic balance of the film matrix. Glycerol concentration (X_3_) had the strongest influence (Equation (3)). As a hygroscopic plasticizer, glycerol increases free volume within the film matrix and also facilitates water uptake through hydrogen bonding, thus enhancing the overall water-holding capacity [17,18]. Sunflower oil, by contrast, had a significant negative linear effect (*p* < 0.05) on EMC, attributed to its hydrophobic character, where the oil reduces the film’s affinity for water by limiting available polar binding sites and forming dispersed non-polar domains [20]. Similar moisture-repelling effects in lipid-incorporated chitosan-based films have been reported by other researchers [21,41].

The influence of gelatin on EMC showed a non-linear, saddle-shaped response (Figure 1B). At intermediate levels of Ge, EMC reached a minimum, suggesting that intermolecular interactions, possibly through polyelectrolyte complex formation between the chitosan and gelatin, occurred at these balanced LCh:Ge ratios [32,42]. Such complexation can reduce the number of free hydroxyl (–OH) and amino (–NH_2_) groups available for binding with water [42]. At either low or high gelatin levels, one of the polymers was present in excess, disrupting optimal interactions and resulting in higher moisture retention. Comparable nonlinear moisture uptake patterns have been noted in chitosan–protein films, where polymer–polymer ratio strongly influences water-binding capacity [37,43], suggesting that the moisture behavior of LCh–Ge matrices is consistent with known polyelectrolyte systems. While no significant interaction terms were identified, the combined nonlinear effects of gelatin and glycerol highlight the need for careful formulation balance to achieve desired moisture resistance.

Degree of swelling (DS) values spanned a wide range (51–230%), reflecting differences in the film’s capacity to absorb water without disintegration. Glycerol (X_3_) was the dominant modulator, showing a strong negative linear effect (*p* < 0.001), which is consistent with its ability to form strong stabilizing hydrogen bonds with both chitosan and gelatin chains, thus promoting matrix cohesion and dimensional stability while restricting water-induced expansion [18,44,45]. However, a significant positive quadratic effect for glycerol (*p* < 0.001) indicated that at high concentrations (40% *w*/*w* polymer), excess unbound glycerol (beyond what is needed for plasticization and interaction with polymer chains) may contribute to renewed water uptake by enhancing the overall hydrophilicity of the film matrix [18].

Gelatin proportion (X_1_) displayed a positive linear effect on DS (*p* < 0.05), attributed to its hydrophilic amino acid residues and tendency to reduce matrix crystallinity when blended with chitosan, thereby facilitating water absorption [32,46]. Conversely, sunflower oil (X_2_) had a significant negative linear effect (*p* < 0.05), acting as a barrier to water ingress through hydrophobic domain formation [47]. Notably, a significant positive interaction between oil and glycerol (X_2_X_3_, < 0.05) was observed for DS (Equation (4)). This suggests that the oil might disrupt the matrix cohesion and dimensional stabilization typically afforded by glycerol at lower levels, potentially by creating phase separations or interfaces where glycerol can accumulate. This could allow glycerol to migrate more freely within the polymer network at these interfaces, thereby promoting localized hydration and swelling, somewhat offsetting the anti-swelling effects that each component might otherwise exhibit. A similar effect of lipid incorporation on the swelling behavior of glycerol-plasticized pea-protein films can be observed in a previous study by Kowalczyk et al. (2024), where glycerol stabilizes chain packing, but lipids introduce heterogeneities that can locally promote water uptake [48]. Such complex nonlinearities observed in the present work are thus characteristic of multi-additive matrices.

Water solubility (WS) ranged from 14% to 32%. The model for WS (Equation (5)) identified all terms as statistically significant except for the quadratic term for oil content. Gelatin content (X_1_) exhibited a strong positive linear effect (*p* < 0.001), indicating higher water solubility with increasing gelatin proportion. This is expected given gelatin’s hydrophilic nature and lower molecular weight compared to chitosan [16,32]. However, a significant negative quadratic term (*p* < 0.05) suggested that solubility declined slightly at high gelatin concentrations (Ge > 50%). This could reflect increased self-association or denser packing of gelatin chains (also reflected in film thickness), which might reduce the availability of free polar groups and therefore resist solubilization.

Like DS, Glycerol (X_3_) exhibited a strong negative linear effect on WS (*p* < 0.001), suggesting that matrix stabilization through hydrogen bonding outweighed its intrinsic hydrophilicity [28,44]. A slight positive quadratic effect (*p* < 0.05) indicated a saturation point beyond which increasing glycerol levels would have diminishing returns. Sunflower oil (X_2_) also reduced WS significantly through its hydrophobic influence, creating phase-separated domains that hinder water diffusion [20,41]. Interestingly, the interaction term between oil and glycerol (X_2_X_3_) was positive, suggesting that the combination of both at high levels can offset their individual solubility-suppressing effects, possibly due to microstructural rearrangements as discussed in the case of DS. In contrast, gelatin–oil (X_1_X_2_) and gelatin–glycerol (X_1_X_3_) interactions had negative coefficients, suggesting that the inclusion of oil or glycerol in gelatin-rich films further limits solubility, possibly by reducing gelatin’s accessibility to water with hydrophobic domains or competitive hydrogen bonding [49,50].

From a functional standpoint, these physical properties are interrelated and must be jointly optimized depending on the target application. Increased FT generally improves mechanical strength and barrier properties but may reduce flexibility and transparency. EMC, DS, and WS collectively influence water interaction dynamics and must be balanced to match environmental conditions and product requirements. For example, low EMC and low DS are highly desirable for packaging high-moisture foods (e.g., fresh meats, cheeses) to maintain dimensional stability of the packaging and prevent microbial growth exacerbated by excess moisture. Conversely, higher WS may be a beneficial attribute in applications such as dissolvable wraps for single-serve food portions or edible pouches for instant soup or beverage mixes, where rapid disintegration in water is desired.

### 3.3. Influence of Formulation Variables on Water Vapour Permeability and Surface Hydrophobicity

Water vapor permeability (WVP) and surface hydrophobicity (assessed through surface contact angle—CA) are critical determinants of an edible film’s effectiveness in protecting moisture-sensitive foods. A lower WVP indicates greater resistance to moisture transfer through the film matrix, while a higher CA reflects greater surface hydrophobicity, which repels liquid water and minimizes wetting [22]. These two properties are often inversely related, as hydrophobic surfaces exhibit lower affinity for water molecules and reduce vapor diffusion. Together, they govern the film’s ability to preserve product quality and shelf life, particularly for perishable items such as dairy, bakery goods, and fresh produce.

The WVP of the composite films ranged from 0.61 to 2.44 g·mm/kPa·h·m^2^, consistent with previously reported values for chitosan-based edible films [16,18,22], confirming that crude lobster chitosan behaves comparably to highly refined commercial chitosan in barrier performance. Glycerol (X_3_) had the most pronounced effect, showing a significant positive linear (*p* < 0.001) and quadratic effect (*p* < 0.05). As a hydrophilic plasticizer, glycerol increases the free volume between polymer chains, promotes water sorption, and facilitates diffusion pathways for water vapor molecules [17,19]. This behavior was pronounced at higher glycerol concentrations (Figure 2B), where increased availability of free glycerol molecules further elevated film hydrophilicity, thereby increasing WVP, a trend reflected in parallel increases in EMC, DS and WS.

Sunflower oil (X_2_) had a negative quadratic effect (*p* < 0.05) on WVP, and films containing ≥10% oil showed markedly lower permeability. This agrees with the literature and is attributed to dispersed oil droplets introducing non-polar domains and creating tortuous diffusion pathways that hinder vapor transport [19,22,41]. At lower concentrations, however, the slight initial increase in WVP may be due to incomplete barrier formation or increased chain spacing and void volume, which offsets the hydrophobic effect [51]. This trend suggests that beyond a threshold, oil becomes more effective at impeding vapor transport, likely due to better distribution and coalescence into stable hydrophobic domains.

Gelatin (X_1_) exhibited a modest but statistically significant negative linear effect (*p* < 0.05), where higher gelatin content slightly reduced WVP. Although gelatin is hydrophilic, its ability to enhance matrix packing and reduce free volume appears to dominate, thereby limiting vapor permeability [32,52]. This aligns with our earlier observations on the impact of gelatin on FT and EMC. These results indicate that despite having hydrophilic components in the film, a denser film with lower moisture content exhibits better moisture barrier performance [9].

A low vapor permeability can be extremely critical for packaging systems for foods that require high resistance to moisture migration. The lowest WVP value observed for the present system was 0.61 g·mm/kPa·h·m^2^, which, while comparable to other chitosan-gelatin films incorporating essential oils [53], is still orders of magnitude higher than petroleum-based standards. Based on WVP, packaging films can be classified as a ‘Good barrier’ (< 0.0042 g·mm/kPa·h·m^2^), ‘Moderate barrier’ (0.0042–0.42 g·mm/kPa·h·m^2^), or a ‘Poor barrier’ (0.42–4.2 g·mm/kPa·h·m^2^). Standard LDPE and HDPE films are ‘Good barriers’, with WVP values of approximately 0.0040 and 0.0012 g·mm/kPa·h·m^2^, respectively [54]. All the tested formulations in the present study are in the ‘Poor barrier’ category, highlighting that this hydrophilic biopolymer system, even with oil, is not a suitable replacement for applications requiring a high moisture barrier performance.

The contact angle (CA) values of the films ranged from ~37° (highly hydrophilic) to ~70° (moderately hydrophobic), reflecting significant variation in surface wettability. Oil content (X_2_) had the strongest influence on CA, exhibiting a significant positive linear effect (*p* < 0.05) and a strong negative quadratic effect (*p* < 0.001). Initial oil incorporation (up to 10%) increased CA by introducing hydrophobic lipid domains on the surface [22,26]. At higher concentrations of oil (> 10%), however, CA started to drop. While the reason for this is unclear, this decrease in hydrophobicity at high lipid loads could be attributed to the correspondingly high concentration of emulsifier (Tween-20) required to stabilize the emulsion. High concentrations of hydrophilic surfactants, such as Tween-20, are known to negatively impact film properties, increase water vapor permeability, and decrease the water contact angle by increasing surface wettability by introducing hydrophilic moieties to the surface [17,55]. Furthermore, this ‘turning point’ in wettability corresponds with observations that excessive oil content can alter film microstructure from ‘homogeneous and smooth’ to ‘heterogeneous and rough’. This structural disorganization, driven by lipid phase separation, leads to weakened contact angle properties [56].

Gelatin also had a complex influence on CA, with a significant negative linear effect (*p* < 0.001) and a positive quadratic term (*p* < 0.05). At high concentrations (>50%), gelatin’s polar amino acid residues likely dominate the film surface, increasing wettability and decreasing CA. At mid-level concentrations (around 50%), however, potential chitosan–gelatin complexation could mask hydrophilic functional groups, maintaining relatively stable CA values [14]. These trends are consistent with other studies that reported similar gelatin-dependent effects on surface polarity [14,57]. Increasing glycerol content significantly reduced CA (*p* < 0.001), which is consistent with its hydrophilic nature and its tendency to increase surface wettability [58].

### 3.4. Influence of Formulation Variables on Optical Properties

Optical properties, particularly light barrier effectiveness and visual clarity, are key considerations in the design and application of edible packaging films, as they influence both food preservation and consumer appeal. The ability of a film to act as a light barrier is especially important for protecting food components, such as lipids, vitamins, and pigments, from photo-oxidation induced by ultraviolet (UV) radiation [16,59,60]. As well, visual clarity and transparency are desirable attributes for products where visibility enhances marketability, such as fresh produce, ready-to-eat meals, and confectionery [9,16,28]. Thus, balancing light protection with aesthetic appeal is often a crucial formulation goal.

The optical responses of the LCh-Ge films varied significantly across formulations. UV opacity (OP_UV_, 230–400 nm) ranged from 550 to 2870 A/mm, while visible opacity (OP_VIS_, 400–800 nm) ranged from 290 to 813 A/mm. Interestingly, glycerol (X_3_) had no statistically significant influence on either OP_UV_ or OP_VIS_, which is consistent with the results from other studies that showed glycerol acting primarily as a plasticizer without significantly altering optical transmission [28,61].

**Figure 3:**
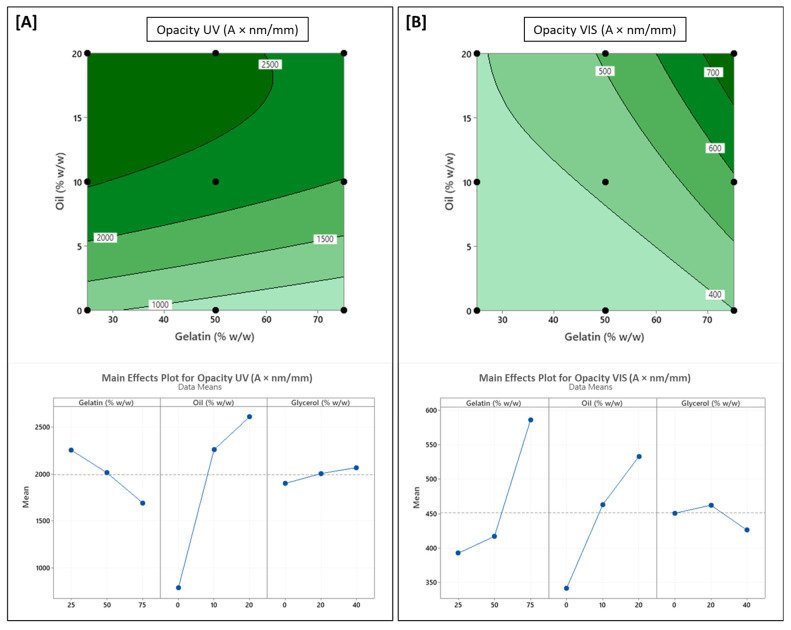
Contour and main effect plots for (**A**) film opacity in the UV spectrum (OP_UV_); (**B**) film opacity in the visible spectrum (OP_VIS_). In each case, the third variable (Glycerol) was fixed at its midpoint value (20% *w*/*w*) to generate the contour plots.

Sunflower oil (X_2_) was the dominant variable affecting UV opacity. Both its linear and quadratic effects were highly significant (*p* < 0.001). Films containing up to 10% oil exhibited a pronounced increase in OP_UV_, attributed to the presence of well-dispersed oil droplets that disrupt light transmission and localized scattering, especially in the UV range of 230–350 nm [20,22]. However, beyond this threshold, increasing oil concentration to 20% did not lead to further gains in UV-blocking performance (Figure 3A), indicating a saturation effect. This plateau is likely caused by droplet coalescence or aggregation at higher oil loadings as larger oil domains become less efficient at scattering shorter UV wavelengths. A previous study on hydroxypropyl-methylcellulose (HPMC) films incorporated with fatty acids observed an increase in film opacity in direct correlation with an increase in the size of the lipid aggregates [62]. This supports the hypothesis that at high concentrations, the oil is no longer acting as small, discrete droplets but as larger, coalesced domains. In contrast, gelatin content (X_1_) exerted a mild but statistically significant negative linear effect on OP_UV_ (*p* < 0.05).

Visible opacity (OP_VIS_) was governed by a more complex interplay of formulation variables. Both gelatin and oil contents had significant linear and quadratic effects (*p* < 0.05), with an additional significant interaction term (X_1_X_2_, *p* < 0.05) indicating that their combined influence was not simply additive (Equation (9)). At low gelatin levels (25% *w*/*w*), oil addition had minimal impact on visible opacity (Figure 3B). However, at higher gelatin levels (≥50% *w*/*w*), increasing the oil content resulted in a near-linear increase in OP_VIS_. This pattern suggests a context-dependent role for gelatin in modulating OP_VIS_. While gelatin alone promotes film clarity due to its low inherent opacity, its structural interactions with oil at higher concentrations can lead to increased haze, likely due to the development of microstructural discontinuities [41,48]. This hypothesis is supported by a previous study by Bertan et al. (2005) on gelatin-lipid films, where increased opacity was directly linked to the occurrence of ‘phase separation’ [63].

This phase separation is also likely triggered by emulsion destabilization. In this ternary system, high gelatin and oil content necessitate a low concentration of chitosan, the system’s primary polycationic stabilizer. Research shows that emulsion stability fails below a minimum critical chitosan concentration, leading to flocculation [64], thus allowing oil droplets to aggregate and scatter more visible wavelengths.

### 3.5. Influence of Formulation Variables on Mechanical Properties

The mechanical properties of edible films are critical for their performance and practical usability in food packaging, as these properties govern their ability to withstand mechanical stress during processing, handling, and storage, maintain structural integrity, and adapt to different packaging formats and food product shapes [20,61]. Tensile strength (TS) ranged broadly across formulations (13.5–79.3 MPa) and was significantly influenced by all three independent variables (Equation (10)). This broad range is highly significant; while the weakest formulations were mechanically poor, the strongest formulations (40–70 MPa) demonstrated a TS superior to LDPE/HDPE (10–20 MPa) and competitive with PET (40–60 MPa), while maintaining a relatively high EAB (20–60%) [23,65,66]. In comparison, most other chitosan-gelatin films reported in the literature often show much lower strength (7–30 MPa) or are noted as being strong but brittle (EAB < 10%) [14,16,41]. These results suggest that the crude chitosan–gelatin matrix is mechanically robust and viable for applications where strength is a key requirement.

Glycerol (X_3_) had the most pronounced weakening effect (*p* < 0.001), consistent with its plasticizing function, which disrupts inter-polymer hydrogen bonding and increases chain mobility, leading to reduced matrix rigidity [17,18]. This decline in TS was steep up to mid glycerol levels (20% *w*/*w*), after which the reduction became less intense, as reflected in the significant positive quadratic term for glycerol (*p* < 0.05).

**Figure 4:**
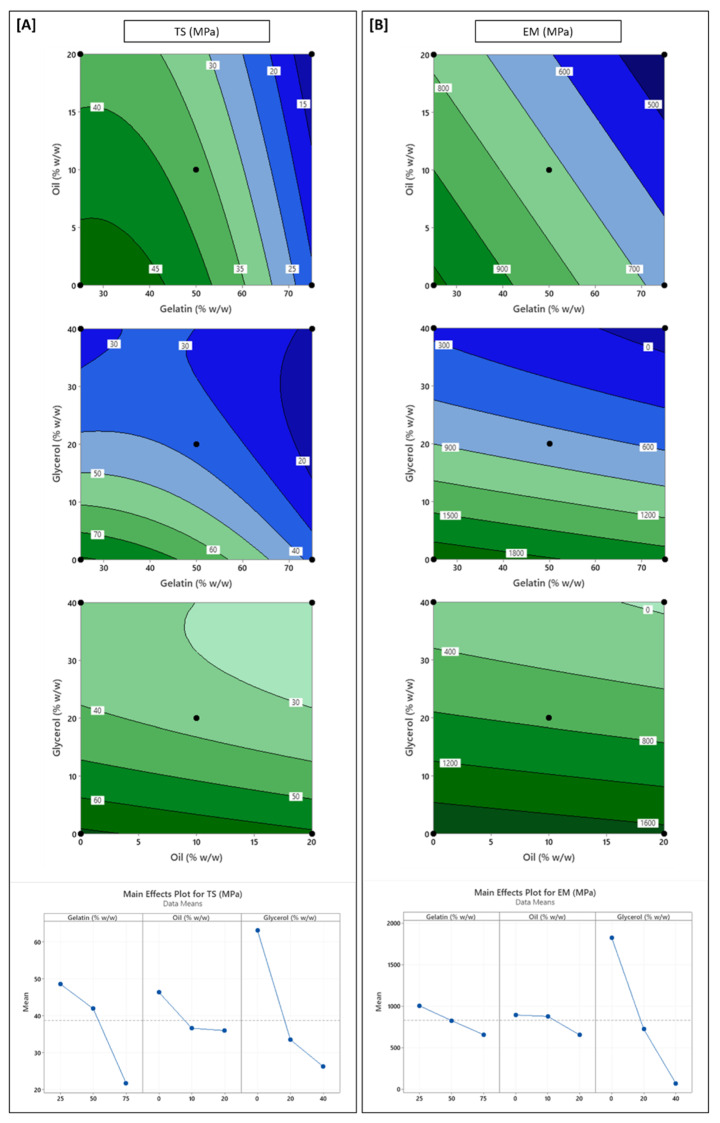
Contour and main effect plots for (**A**) tensile strength (TS); (**B**) elastic modulus (EM). In each case, the third variable was fixed at its midpoint value to generate the contour plots.

Gelatin (X_1_) also significantly reduced TS, particularly at higher levels (> 50%), as evident by a positive linear but a negative quadratic term (Equation (10), Figure 4A). Indeed, films comprising only gelatin have characteristically low TS and rigidity offered by their neat films [32,61]. While gelatin–chitosan interactions initially help maintain matrix strength at low gelatin content, the dilution of chitosan at higher gelatin ratios limits interpolymer interactions, thereby decreasing film cohesion and strength [32]. A positive interaction between gelatin and glycerol (X_1_X_3_, *p* < 0.05) was also observed, suggesting that the negative impact of glycerol on TS was partially mitigated in the presence of gelatin. This may result from additional hydrogen bonding and potential crosslinking between the two components at higher glycerol levels, which supports the matrix structure despite reduced polymer–polymer cohesion.

Oil content (X_2_) also contributed to a statistically significant (*p* < 0.05) decrease in TS, though the effect was less intense compared to glycerol or gelatin. This can be attributed to the introduction of non-polar domains that disrupt cohesive hydrogen bonding among polymer chains and increase intermolecular spacing, also evident from increased film thickness in oil-rich formulations [14,20,41]. Similar mechanical weakening from lipids has been widely documented in chitosan–oil composites [21,41,67,68], attributed to the disruption of intermolecular hydrogen bonding. The effect of oil became more pronounced in gelatin-rich films (Figure 4A), possibly due to reduced chitosan content, which weakened emulsification and allowed oil droplets to phase separate or aggregate, further reducing film integrity.

Elastic modulus (EM), a measure of film stiffness, followed trends largely similar to TS (Figure 4B). As expected, glycerol exhibited a strong negative linear response (*p* < 0.001), significantly softening the matrix through plasticization and enhancing molecular mobility. A positive quadratic effect of glycerol (*p* < 0.05) further indicated a diminishing marginal reduction in stiffness at higher glycerol levels, again suggesting a limit to the softening effect. Gelatin and oil both exhibited negative linear effects on EM (*p* < 0.05), albeit with smaller magnitudes. While gelatin’s influence is associated with the relatively low inherent stiffness compared to chitosan [16], oil, like glycerol, reduces molecular cohesion by introducing non-polar domains, leading to a decrease in matrix rigidity [41].

Elongation at break (%EAB) was influenced solely by glycerol content (Equation (11)). Glycerol exhibited a significant positive linear effect on EAB (*p* < 0.001), confirming its role as a primary plasticizer that enhances chain mobility and allows films to stretch extensively before rupture [44]. EAB values increased steadily with glycerol content, reaching up to 100% in the most plasticized formulations. Neither oil nor gelatin had a significant main effect on EAB (*p* > 0.05). Although some previous studies have reported mild plasticizing effects of edible oils on chitosan-based films [20,22], oil did not significantly influence EAB in the present study. This could be due to the nature of oil dispersion in the matrix. Sunflower oil exists as discrete droplets rather than a continuous phase. These droplets, even if emulsified, may not integrate sufficiently with the polymer network to reduce cohesive forces at a molecular level. Moreover, if droplet coalescence occurs at higher oil contents, oil domains may act more as voids or stress concentrators than as plasticizers, reducing their capacity to enhance extensibility. Thus, the overall impact of oil on flexibility was limited and inconsistent across the tested range. These results are consistent with some other studies showing that lipids rarely modulate extensibility in chitosan systems unless they form continuous phases or integrate molecularly with the polysaccharide network [7,20,21,22].

### 3.6. Summary of Critical Film Formulation Trade-Offs

Taken together, these film property results highlight a series of critical formulation trade-offs, revealing the complex and often conflicting roles of each component which require a balanced optimization for targeted applications. Glycerol acts as the primary plasticizer, essential for imparting mechanical flexibility by dramatically increasing elongation at break (%EAB). This benefit comes at the cost of significantly compromising mechanical strength (TS) and stiffness (EM), as well as moisture resistance, by increasing bulk film hydrophilicity (higher EMC and WVP) and surface wettability (lower CA). Sunflower oil is the key hydrophobic and light-protective agent; it improves moisture barrier properties by lowering WVP and water solubility and is the dominant factor in providing a UV-protective barrier. However, these advantages are offset by a reduction in mechanical strength and a decrease in visual clarity, as oil increases haze. Gelatin plays the most transitional role, enhancing film density which can reduce WVP, but its hydrophilic nature increases water solubility and swelling. Mechanically, it weakens the film at higher concentrations, and it can interact with oil to reduce transparency. These competing effects underscore that the final functionality of LCh-Ge composite films depends on the precise, synergistic balance of its ingredients, enabling the creation of diverse materials. The ability to modulate film properties by adjusting the concentrations of gelatin, oil, and glycerol highlights the versatility of this composite system for tailored packaging design. Similar multidirectional trade-offs among plasticizer, protein, and lipid components have been reported across chitosan–gelatin and chitosan–plant oil studies [19,23,26,37,69,70], reinforcing the need for RSM-guided optimization to navigate inherently conflicting design requirements.

### 3.7. Optimization of Films and Validation of Regression Models for Specific Food Packaging Applications

The fitted RSM regression models were used to optimize the film formulation across multiple performance attributes. This multivariate strategy enabled the tailoring of the films to meet the functional requirements of specific food packaging applications by identifying different optimal concentrations of fish gelatin, sunflower oil, and glycerol in lobster chitosan-based film-forming dispersions (FFDs). The optimization focused on attributes directly affecting film functionality, such as mechanical performance, water interaction behavior, optical clarity, and moisture barrier properties. Table 3 presents the three optimized formulations, including their component ratios and overall desirability scores, each tailored to a distinct application context.

To assess the predictive reliability of the optimized models, all three distinct formulations (F1, F2 and F3) were prepared and experimentally evaluated for the targeted response parameters. The results demonstrated excellent agreement between predicted and measured values, with all experimental responses falling within the 95% confidence intervals of their respective model estimates (Table 4). Residual deviations were generally below 5%, affirming the robustness and accuracy of the developed RSM models.

The first optimized formulation (F1; desirability = 0.81) was designed for applicability in food products with moist surfaces, such as cheese or fresh-cut meats. It was optimized for low WS and DS to limit film degradation in the presence of surface moisture, high contact angle to enhance surface hydrophobicity, and low WVP and high UV opacity to reduce moisture ingress and protect the product from photodegradation. However, a critical analysis of the validated data (Table 4) reveals a mismatch for this ‘real food application’. The achieved WVP of 1.05 g·mm/kPa·h·m^2^ is classified as a ‘Poor’ moisture barrier and is over 260 times more permeable than LDPE, making it unsuitable for preventing moisture loss in high-moisture foods [54]. The second formulation (F2), which yielded the highest desirability score (0.84), was intended for packaging relatively dry yet irregularly shaped produce, such as mushrooms or cut fruits. Its design prioritized high flexibility (EAB), excellent transparency, and strong UV protection to accommodate mechanical demands and maintain product visibility. Since the surface of such products contains little free moisture, parameters like DS, WS, and CA were not included in this optimization. This application is strongly supported by the data. The film’s TS (41 MPa) is superior to LDPE (10–30 MPa) and does not compromise much on EAB (49%), thus providing excellent structural integrity. Furthermore, its ‘Poor’ WVP, a failure for F1, is a feature for this application. Respiring produce like fruits and mushrooms requires a semi-permeable film to allow gas exchange and prevent moisture buildup, which leads to anaerobic spoilage. The third formulation (F3; desirability = 0.77) was tailored for dry food applications, such as edible films for instant noodles or soup bases. It was optimized for high WS and DS to facilitate rapid dissolution in hot water, while mechanical and optical parameters were deprioritized, given the assumption of secondary protective packaging. This application is also well-supported. A key finding is that this highly soluble film (31% WS) retained a very high mechanical strength (TS of 49 MPa), a combination that is highly practical for a dissolvable pouch that must survive transport and handling before use.

### 3.8. Antimicrobial Activity of Optimized Films

**Figure 5:**
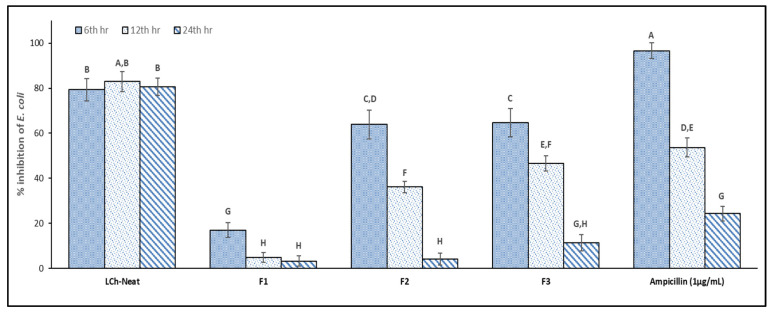
Antimicrobial activity (% inhibition of *E. coli*) for neat LCh and optimized LCh-Ge films with LDPE films as the control (0% inhibition). Significantly different means (*p* < 0.005) are indicated with different letters.

To assess their potential for active packaging, the neat LCh and optimized LCh-Ge composite films were tested for antimicrobial activity against *Escherichia coli*. The results shown in Table 5 and Figure 5 reveal that neat LCh films exhibited potent biocidal activity, achieving up to 80% inhibition over a 24-h period, an efficacy comparable or superior to the ampicillin positive control at different time-points. This well-documented activity is attributed to chitosan’s polycationic nature; its protonated amino groups interact electrostatically with negatively charged microbial cell membranes, disrupting their integrity and causing cell death [7,57,71]. Consequently, the availability of these free amino groups dictates the film’s antimicrobial potential.

While all three optimized composite formulations (F1, F2 and F3) exhibited measurable inhibition, their activity was significantly lower than that of the neat LCh films. This reduction may primarily be due to two factors: the lower overall chitosan concentration in the composite matrix and molecular interactions between chitosan, gelatin, and glycerol [71,72,73]. These strong hydrophilic interactions can bind and shield chitosan’s bioactive amino groups, limiting their availability to interact with bacterial cells, an observation consistent with previous studies on chitosan-gelatin composites [32,71,72,74].

Among the composites, formulations F2 and F3 showed higher inhibitory activity than F1. The superior performance of F2 can be linked to its higher chitosan content (56% *w*/*w*) compared to F1 (40% *w*/*w*). F3’s effectiveness could be due to its comparatively high water-solubility and low glycerol content, which may have facilitated a greater release of active chitosan molecules into the growth media [32]. Interestingly, after 24 h, the inhibition for F1 and F2 films became negligible. This suggests that over time, gelatin dissolved from the films may have been utilized as a nutrient source by the bacteria, counteracting the initial inhibitory effect. These results highlight a fundamental formulation trade-off: additives like gelatin and glycerol, while necessary for optimizing mechanical and physical properties, compromise chitosan’s antimicrobial efficacy. Although the residual bioactivity supports the films’ potential for applications requiring short-term microbial inhibition, future work could focus on incorporating other natural antimicrobials such as essential oils to enhance this function. Moreover, to establish their real-world applicability, antimicrobial performance should be assessed on real food systems where surface chemistry and moisture levels differ markedly from broth culture conditions.

## 4. Conclusions

This study successfully developed empirical optimization models for biodegradable edible films formulated from crude lobster-shell chitosan, fish gelatin, sunflower oil, and glycerol, using RSM. The experimental design allowed the analysis of complex interactions among components and the identification of key trade-offs between competing film attributes such as mechanical strength, water sensitivity, transparency, and barrier performance. Results demonstrated that film functionality is governed by nonlinear, synergistic, and antagonistic effects, underscoring the need for compositional tuning. Simultaneous multi-response optimization produced balanced formulations suited for diverse food packaging applications. Specifically, three distinct formulations were identified: F1, optimized for moisture resistance (59.3% Ge, 11.3% O, 14.9% Gly); F2, optimized for high flexibility (43.8% Ge, 15.4% O, 17.4% Gly); and F3, optimized for high water-solubility (59.7% Ge, 0% O, 11.6% Gly). Experimental validation of these formulations confirmed the predictive reliability of the RSM models under controlled conditions. A critical comparison of these optimized films against petroleum-based standards revealed both the strengths and weaknesses of the developed system for real-world applications. Formulations F2 and F3 demonstrated exceptional mechanical strength (41 MPa and 49 MPa, respectively), competitive with or superior to conventional LDPE (10–30 MPa). This validated their potential for specific ‘real food applications’, such as F2 as a semi-permeable, flexible wrap for respiring produce and F3 as a high-strength, water-soluble pouch. Conversely, the high-barrier formulation (F1) proved unsuitable for moist-food applications, as its WVP (1.05 g·mm/kPa·h·m^2^) was orders of magnitude higher than LDPE (~0.004 g·mm/kPa·h·m^2^). Although antimicrobial activity decreased in composite films, likely due to interactions with additives, residual inhibition against *E. coli* was retained, supporting potential applications in active packaging for perishable foods. A major contribution of this work is the valorization of marine processing waste. The use of minimally processed lobster-shell chitosan presents a sustainable, low-cost alternative to commercial chitosan, without compromising film functionality. This integrated approach—combining waste utilization, empirical modeling, and functional evaluation—advances the development of high-performance biopolymer films aligned with circular economy principles. While the findings are promising, testing was limited to static laboratory conditions. Future work should assess film behavior under real-world storage conditions, evaluate sensory properties, and investigate the incorporation of natural antimicrobials or antioxidants. Consumer acceptance, life-cycle assessment, and cost–benefit analyses will be essential to support industrial translation of these sustainable packaging materials.

## Figures and Tables

**Table 2:** Statistical significance of regression models and individual terms for each film property investigated, as represented by the F values.

Source	DF ^#^	FT	EMC	DS	WS	OP_UV_	OP_VIS_	TS	% EAB	EM	WVP	CA
Full Model	9	48.26 **	41.19 **	16.11 *	48.94 **	9.15 *	40.79 **	37.91 **	18.65 **	68.88 **	29.75 **	16.61 *
Linear	3	132.04 **	112.43 **	30.23 **	73.06 **	19.09 *	102.41 **	89.61 **	54.22 **	197.59 **	78.90 **	22.55 *
Ge (X_1_)	1	80.51 **	10.01 *	6.79 *	43.47 **	28.74 *	26.56 *	88.63 **	3.19	22.22 *	6.56 *	37.14 **
O (X_2_)	1	298.81 **	14.77 *	14.45 *	52.40 **	28.08 *	278.28 **	13.24 *	0.27	10.56 *	6.30 *	6.33 *
Gly (X_3_)	1	16.81 *	312.49 **	69.44 **	123.32 **	0.46	2.39	166.96 **	159.18 **	559.99 **	223.82 **	26.19 **
Square	3	6.49 *	10.58 *	13.49 *	57.65 **	3.69	18.67 *	17.18 *	0.05	8.23 *	9.20 *	24.74 **
Ge * Ge	1	2.33	10.54 *	0.8	166.97 **	9.20 *	0.01	15.06 *	0.09	0.01	0.29	15.07 *
O * O	1	17.75 *	1.46	0.45	3.7	1.28	55.68 **	4.9	0.02	5.24	13.93 *	55.34 **
Gly * Gly	1	0.1	19.00 *	37.84 **	7.36 *	1.14	0.02	33.31 *	0.06	20.46 *	14.83 *	1.88
Interaction	3	6.26 *	0.56	4.60 *	16.09 *	4.66 *	1.3	6.95 *	1.69	0.82	1.16	2.55
Ge * O	1	3.12	0.02	0.02	16.48 *	13.24 *	0.11	2.01	0.01	0.02	0.43	1.01
Ge * Gly	1	8.31 *	0.2	0.56	11.38 *	0.07	0.26	17.94 *	4.16	0.84	0.02	4.34
O * Gly	1	7.35 *	1.49	13.21 *	20.42 *	0.66	3.53	0.91	0.92	1.61	3.02	2.31
Lack of Fit	3	0.83	1.93	5.04	0.68	1.74	0.32	3.04	5.23	0.78	2.05	1.67

^#^ degrees of freedom; * statistically significant at *p* < 0.05; ** statistically significant at *p* < 0.001; FT: film thickness; EMC: equilibrated moisture content; DS: degree of swelling; WS: water solubility; OP_VIS_ and OP_UV_: film opacity in the visible and UV spectrum; TS: tensile strength; EAB: elongation at break; EM: elastic modulus; WVP: water vapour permeability; CA: surface contact angle.

**Table 3:** Examples of three optimized composite film formulations for specific applications based on different target responses.

Response Parameters	Formulation 1	Formulation 2	Formulation 3
	Target	Target	Target
FT (µm)	NO	NO	NO
EMC (%)	NO	NO	NO
DS (%)	Minimize	NO	Maximize
WS (%)	Minimize	NO	Maximize
OP_VIS_ (A × nm/mm)	Minimize	Minimize	NO
OP_UV_ (A × nm/mm)	Maximize	Maximize	NO
TS (MPa)	Range (35–45)	Range (35–45)	Range (35–45)
EAB (%)	Range (35–45)	Range (45–55)	Range (35–45)
EM (MPa)	Range (800–1000)	Range (800–1000)	Range (800–1000)
WVP (g × mm/kPa × h × m^2^)	Minimize	Minimize	Minimize
CA (°)	Maximize	NO	NO
**Ge (X_1_)** (% w/w polymer)	59.34	43.76	59.69
**O (X_2_)** (% w/w polymer)	11.31	15.42	0
**Gly (X_3_)** (% w/w polymer)	14.94	17.41	11.55
**Desirability (D)**	0.81	0.84	0.77

NO: Not optimized; Ge: fish gelatin proportion; O: sunflower oil content; Gly: glycerol content; FT: film thickness; EMC: equilibrated moisture content; DS: degree of swelling; WS: water solubility; OP_VIS_ and OP_UV_: film opacity in the visible and UV spectrum; TS: tensile strength; EAB: elongation at break; EM: elastic modulus; WVP: water vapour permeability; CA: surface contact angle.

**Table 4:** Predicted and experimental response data for three optimized film formulations.

Response Parameters	Formulation 1		Formulation 2		Formulation 3	
	Predicted Value and Range at 95% CI *	Experimental Value	Predicted Value and Range at 95% CI *	Experimental Value	Predicted Value and Range at 95% CI *	Experimental Value
FT (µm)	64.79 (62.75–66.84)	66.87 ± 4.61	76.01 (74.14–77.87)	74.53 ± 5.37	52.12 (48.89–55.34)	51.12 ± 7.69
EMC (%)	10.01 (8.74–11.25)	12.25 ± 1.36	11.06 (9.65–12.48)	13.12 ± 0.92	11.11 (9.42–12.79)	11.40 ± 1.45
DS (%)	86.53 (75.23–97.83)	83.51 ± 5.47	59.14 (46.58–71.71)	61.06 ± 2.04	138.18 (121.03–155.34)	157.21 ± 4.89
WS (%)	23.74 (22.85–24.61)	23.02 ± 1.81	21.61 (20.61–22.62)	19.98 ± 1.27	28.59 (27.24–29.95)	30.87 ± 3.35
OP_VIS_ (A × nm/mm)	480.46 (446.35–514.58)	498.80 ± 23.89	436.74 (396.32–477.17)	438.59 ± 9.49	333.78 (279.62–387.95)	297.00 ± 7.73
OP_UV_ (A × nm/mm)	2262.4 (2142.3–2382.5)	2176.1 ± 39.2	2656.0 (2543.9–2768.2)	2683.9 ± 53.6	679.6 (504.9–854.3)	593.9 ± 28.3
TS (MPa)	34.63 (30.39–38.86)	33.08 ± 2.41	39.69 (34.94–44.44)	41.01 ± 3.12	44.07 (38.36–49.79)	48.79 ± 5.59
EAB (%)	40.94 (36.01–45.88)	41.39 ± 10.05	45.77 (41.05–50.48)	48.71 ± 13.74	34.32 (28.88–39.76)	27.12 ± 6.34
EM (MPa)	880.4 (791.1–969.7)	863.5 ± 51.2	821.1 (722.0–920.1)	798.6 ± 43.5	1187.5 (1062.7–1312.4)	1259.6 ± 69.1
WVP (g × mm/kPa × h × m^2^)	1.06 (0.94–1.18)	1.05 ± 0.03	1.09 (0.98–1.21)	1.11 ± 0.04	0.86 (0.68–1.02)	0.79 ± 0.06
CA (°)	61.8 (58.3–65.2)	58.9 ± 2.5	63.6 (60.2–66.9)	61.8 ± 1.9	48.5 (43.6–53.4)	45.6 ± 1.6

CI *: confidence interval; FT: film thickness; EMC: equilibrated moisture content; DS: degree of swelling; WS: water solubility; OP_VIS_ and OP_UV_: film opacity in the visible and UV spectrum; TS: tensile strength; EAB: elongation at break; EM: elastic modulus; WVP: water vapour permeability; CA: surface contact angle.

**Table 5:** Antimicrobial testing results for LCh-based films (neat and optimized) and control samples, showing the *E. coli* remaining after 24 h of incubation.

Samples	Log (CFU/mL)
LDPE	10.11 ± 0.18 ^A^
LCh-Neat	6.20 ± 0.19 ^C^
F1	10.03 ± 0.11 ^A,B^
F2	9.97 ± 0.15 ^A,B^
F3	9.66 ± 0.07 ^A,B^
Ampicillin (1 µg/mL)	9.53 ± 0.09 ^B^

LDPE: low-density polyethylene; LCh: lobster-shell chitosan; F: formulation. Results with the same superscript letter indicate that the difference in mean values is statistically insignificant (*p* > 0.05) as determined by Tukey’s HSD test.

## Data Availability

The raw data supporting the conclusions of this article will be made available by the authors upon request.

## Supplementary Materials

The following supporting information can be downloaded at: https://www.mdpi.com/article/10.3390/polym17233132/s1, Table S1: Experimental mean data for all film property parameters for the tested film formulations; Table S2: Coefficient of determination (R^2^), Adjusted R^2^, and Predicted R^2^ values for the reduced regression models for all film property responses.
