# Supplementary material for: Development of Tailored Composite Biopolymer Film Formulations Using Minimally Refined Chitosan from American Lobster (Homarus americanus) Shell Waste for Different Food Packaging Applications
Source: Polymers (Basel). 2025 Nov 25;17(23):3132. doi: 10.3390/polym17233132 (PMC12694187; doi:10.3390/polym17233132)
Supplement: Supplementary file 1 [file polymers-17-03132-s001.zip › polymers-3986062-supplementary.pdf]

Supplementary Data

Table S1 - Experimental mean data for all film property parameters for the tested film formulations.

Experimental Run	FT (µm)	EMC (%)	DS (%)	WS (%)	OP_{UV} (A*nm/mm)	OP_{VIS} (A*nm/mm)	TS (MPa)	EAB (%)	EM (MPa)	WVP (g*mm/kPa*h*m^2)	CA (°)
1	64.0	18.2	71.7	17.0	962.7	350.1	44.2	65.0	1031.1	1.28	49.3
2	46.4	15.8	97.9	23.6	548.9	364.4	27.5	40.0	644.3	0.90	38.3
3	89.2	13.6	52.6	16.3	2876.0	427.2	41.5	63.8	659.8	1.06	54.7
4	79.4	11.3	83.9	16.0	2361.4	813.2	13.5	38.0	303.9	0.86	37.2
5	66.3	10.9	145.0	18.1	2499.9	400.3	79.3	20.6	2097.7	0.84	69.9
6	58.4	5.7	187.7	25.8	1919.1	600.9	30.9	6.0	1676.9	0.61	63.0
7	77.7	31.4	63.3	14.1	2677.4	393.3	29.3	78.5	241.0	2.44	61.2
8	57.1	27.6	81.9	16.1	1940.1	567.0	15.1	99.7	11.2	2.25	40.9
9	51.8	9.7	230.0	32.1	557.5	361.7	79.1	7.4	1888.5	0.73	55.9
10	82.6	4.0	101.5	23.8	2620.3	440.2	62.9	10.9	1631.5	0.61	58.4
11	65.5	25.2	75.5	21.8	1084.6	291.7	34.5	96.3	20.4	2.27	43.0
12	84.3	23.3	64.0	21.1	2568.4	453.1	26.0	83.0	28.4	1.67	55.3
13	67.8	15.1	59.1	22.0	1967.0	422.8	33.8	57.5	633.7	1.45	61.7
14	67.4	13.8	70.3	22.3	2234.6	394.4	31.2	48.6	797.7	1.19	59.7
15	64.2	12.0	50.9	23.3	2327.1	446.3	34.1	55.4	711.5	1.41	64.7
16	65.0	12.2	76.0	22.1	2464.5	430.2	38.9	45.2	919.3	1.29	60.9
17	69.9	12.9	64.8	24.2	2291.1	513.6	36.7	48.1	830.0	1.22	66.6

FT: film thickness; EMC: equilibrated moisture content; DS: degree of swelling; WS: water solubility; OP_{UV} and OP_{VIS}: film opacity in the UV and visible spectrum; TS: tensile strength; EAB: elongation at break; EM: elastic modulus; WVP: water vapour permeability; CA: surface contact angle.

Table S2 – Coefficient of determination (R^2), Adjusted R^2, and Predicted R^2 values for the reduced regression models for all film property responses.

	FT	EMC	DS	WS	OP_{UV}	OP_{VIS}	TS	EAB	EM	WVP	CA
R^2	97.14	97.31	94.14	97.61	87.97	96.44	95.75	91.02	97.65	96.08	89.44
$R^2_{adjusted}$	95.43	96.09	91.48	95.22	83.95	95.62	93.21	90.42	96.87	94.31	84.64
$R^2_{predicted}$	91.19	93.04	78.68	86.46	70.01	94.33	81.42	88.63	95.21	89.53	70.84

FT: film thickness; EMC: equilibrated moisture content; DS: degree of swelling; WS: water solubility; OP_{UV} and OP_{VIS}: film opacity in the UV and visible spectrum; TS: tensile strength; EAB: elongation at break; EM: elastic modulus; WVP: water vapour permeability; CA: surface contact angle.
